# Whole-genome sequencing reveals molecular characterization of carbapenem-resistant *Pseudomonas aeruginosa* clinical isolates from a third-tier general hospital in southwest China

**DOI:** 10.3389/fcimb.2026.1843484

**Published:** 2026-05-08

**Authors:** Ping He, Jing Yang, Yi Wang, Qi Chen, Yuyang Xie, Mingxiao Han, Chenhao Zhao, Qiyuan Jin, Haifang Zhang

**Affiliations:** 1 Department of Clinical Laboratory, The Second Affiliated Hospital of Soochow University, Suzhou, Jiangsu, China; 2 Department of Clinical Laboratory, Sichuan Science City Hospital, Mianyang, Sichuan, China; 3 Suzhou Medical College of Soochow University, Suzhou, Jiangsu, China; 4 MOE Key Laboratory of Geriatric Diseases and Immunology, Soochow University, Suzhou, Jiangsu, China

**Keywords:** antibiotic resistance, CRPA, PFGE, ST, WGS

## Abstract

**Objective:**

To characterize the molecular characteristics of carbapenem-resistant *Pseudomonas aeruginosa* (CRPA) from a third-tier general hospital in southwest China.

**Methods:**

A total of 106 non-duplicate clinical isolates of CRPA were collected from January 2019 to December 2023 at Science City Hospital in Sichuan Province, China. Antimicrobial susceptibility testing was performed to characterize the resistance profiles of the isolates. Whole-genome sequencing (WGS) was conducted to comprehensively characterize the sequence types (STs), virulence factors, and resistance genes. Pulsed-field gel electrophoresis (PFGE) was employed to analyze DNA fingerprinting patterns, clarifying the clonal relatedness among strains and tracing potential clonal dissemination.

**Results:**

Antimicrobial susceptibility testing demonstrated that all 106 CRPA isolates were resistant to imipenem. Consistent resistance to cefazolin and cefotetan were observed in 103 isolates. ST640 was identified as the predominant type, along with two novel STs, designated ST4440 and ST4513. Molecular characterization revealed that all isolates carried the efflux pump genes (mexA, mexB), the blaPDC (AmpC enzyme) gene and blaOXA-50, while metallo-β-lactamase genes were detected in only two isolates. All 106 isolates carried virulence gene exoT, with exoY and exoS detected in 98.1% and 91.5%, respectively. PFGE results showed that no predominant clone was observed at the 85% similarity level, suggesting that the isolates were mainly from sporadic infections.

**Conclusion:**

Resistance in CRPA isolates is predominantly mediated by efflux pumps and AmpC enzymes, along with the presence of the blaOXA-50. The observed high genetic diversity underscores the need for enhanced infection control measures.

## 1 Introduction

*Pseudomonas aeruginosa* (PA) is a common opportunistic pathogen responsible for severe respiratory infections, including ventilator-associated pneumonia and chronic infections in patients with cystic fibrosis (Jurado-Martín et al., 2021; Letizia et al., 2025). And its ability to colonize damaged skin and mucosal surfaces can lead to burn wound infections, chronic wound infections, diabetic foot infections, and various superficial infections (Hasannejad-Bibalan et al., 2021; Rashki et al., 2025; Yamberla et al., 2025). In recent years, the antimicrobial resistance of PA has become increasingly prominent, with a continuous rise in the detection rate of multidrug-resistant strains (Al-Orphaly et al., 2021; Rashki et al., 2025). Of particular concern, the reliance on carbapenems in clinical practice, coupled with the scarcity of other effective treatment options, has led to an increasing prevalence of carbapenem-resistant *Pseudomonas aeruginosa* (CRPA), posing significant challenges to clinical anti-infective therapy (Tenover et al., 2022). Furthermore, studies have shown that CRPA infections significantly prolong hospital stays, increase morbidity and mortality rates (Liu et al., 2015; Buehrle et al., 2017; Cai et al., 2017). 

The resistance mechanisms of CRPA to carbapenem antibiotics primarily involve three main mechanisms (Pang et al., 2019). The first is the overexpression of active efflux pump systems, particularly the MexAB-OprM (MexA, MexB, and OprM) pump (Lorusso et al., 2022). MexAB-OprM belongs to the RND (Resistance-Nodulation-Division) family and can recognize and efflux multiple antibiotics, particularly meropenem (Lorusso et al., 2022; Quddus et al., 2023). The second mechanism is the loss or reduced expression of outer membrane porins, leading to decreased drug penetration. Among these, the OprD porin is the main channel for carbapenem antibiotics (especially imipenem) to enter the cell (Li et al., 2012). Mutations, insertions, or downregulation of the *oprD* gene can impair this porin, significantly reducing carbapenems uptake (Richardot et al., 2015; Sherrard et al., 2022). The third mechanism involves the production of carbapenemases, enzymes that directly hydrolyze and inactivate the molecular structure of carbapenem antibiotics (Hammoudi Halat and Ayoub Moubareck, 2022). Based on the Ambler molecular classification system, β-lactamases are divided into classes A, B, C, and D. Enzymes capable of efficiently hydrolyzing carbapenems are mainly distributed among classes A, B, and D (Hammoudi Halat and Ayoub Moubareck, 2022). Although class C or AmpC β-lactamases typically do not affect susceptibility to carbapenems, when AmpC enzymes are overexpressed in conjunction with the loss of the OprD porin, the bacteria can develop resistance to imipenem (Freed et al., 2024). In clinically isolated resistant strains, these three mechanisms often exhibit synergistic effects. For instance, the combination of OprD deficiency and overexpression of the cephalosporinase AmpC can further reduce susceptibility to meropenem and doripenem; similarly, the combination of porin loss and efflux pump overexpression can synergistically enhance multidrug resistance in bacteria (Alimoghadam et al., 2024; Falcone et al., 2024).

Given the complexity and diversity of CRPA resistance mechanisms and the current lack of consensus on an optimal therapeutic regimen, local *in vitro* data on the prevalence of resistance mechanisms, antimicrobial susceptibility testing, and MIC distributions are essential for optimizing antimicrobial selection and dosing. Therefore, this study conducted an in-depth investigation of CRPA isolates collected from Science City Hospital in Sichuan Province between 2019 and 2023, aiming to elucidate their resistance mechanisms and molecular epidemiological characteristics. The findings are expected to provide guidance for the rational selection of antimicrobial agents in clinical practice and to offer fundamental data for resistance surveillance and the formulation of infection control strategies.

## 2 Materials and methods

### 2.1 Strain source, culture and identification

A total of 106 CRPA strains were obtained from patients at Science City Hospital, Sichuan Province, from January 2019 to December 2023. All isolates were cultured on blood agar plates and incubated aerobically at 37 °C with 5% CO_2_ for 18–24 h. Species identification was performed using matrix-assisted laser desorption/ionization time-of-flight mass spectrometry (MALDI-TOF MS). Following purification, the isolates were stored in 30% glycerol at -70 °C for long-term preservation and subsequent analyses.

### 2.2 Antimicrobial susceptibility testing

The VITEK 2 Compact system was employed for antimicrobial susceptibility testing. In brief, a single colony was streaked onto blood agar and incubated at 37 °C with 5% CO_2_ for 18–24 h. AST-GN13 cards were allowed to equilibrate to room temperature for 15–20 min prior to use. Following calibration of the turbidity meter, two sterile tubes containing 3 mL of 0.45% NaCl solution were prepared. Bacterial colonies were suspended in the first tube to achieve a turbidity equivalent to a 0.5-0.63 McFarland standard. Subsequently, 145 μL of this suspension was transferred to the second tube and mixed thoroughly. The test cards were then placed onto a cassette, inserted into the bacterial suspension, and loaded into the VITEK 2 Compact system for automated incubation and interpretation of results.

### 2.3 Modified carbapenem inactivation method test

A pure bacterial colony was suspended in 2 mL of TSB broth. A meropenem disk was added to the suspension and incubated at 37 °C for 4 h. Concurrently, a lawn of *Escherichia coli* ATCC 25922 (0.5 McFarland) was prepared on an MH agar plate. Following incubation, the meropenem disk was retrieved from the suspension and placed onto the inoculated plate, which was then incubated at 37 °C for 18–24 h. Carbapenemase production (positive) was indicated by a zone diameter of 6–15 mm, or 16–18 mm with pinpoint colonies within the zone. A clear zone of ≥19 mm indicated a negative result. *Klebsiella pneumoniae* ATCC BAA-1705 and ATCC BAA-1706 served as positive and negative controls, respectively.

### 2.4 Polymerase chain reaction

The DNA of the strain was extracted separately with the boiling template method, and the porin *oprD* gene was performed through PCR.

### 2.5 Whole-genome sequencing (WGS)

Bacterial genomic DNA was extracted using the OMEGA DNA Extraction Kit following the manufacturer’s instructions. The detailed procedure was as follows: A single colony was inoculated into LB broth containing imipenem and incubated overnight at 37 °C with shaking at 200 r/min. The bacterial culture was centrifuged, and the supernatant was discarded. The pellet was resuspended in 100 μL of TE buffer, and 20 μL of lysozyme was added, followed by incubation at 37 °C for 30 min in a water bath. The suspension was then transferred to a sterile EP tube containing 0.025-0.03 g of glass beads and vigorously vortexed for 5 min. After allowing the beads to settle, the supernatant was collected, and 100 μL of BTL buffer and 20 μL of proteinase K were added, mixed thoroughly, and incubated at 55 °C for 1.5 h. Subsequently, 5 μL of RNase was added, and the mixture was incubated at room temperature for 5 min. After centrifugation, the supernatant was transferred to a new tube, and 220 μL of BDL buffer was added, mixed, and incubated at 65 °C for 10 min. Then, 220 μL of absolute ethanol was added and mixed thoroughly by vortexing. The entire mixture was transferred to a DNA Mini Column and centrifuged to discard the flow-through. The column was washed once with 500 μL of HBC buffer and twice with 700 μL of DNA Wash Buffer. After an additional centrifugation to dry the column, it was incubated at room temperature for 5 min. DNA was eluted by adding 60 μL of preheated ddH_2_O (65 °C) to the center of the column, incubating briefly, and centrifuging. The DNA quality was assessed using a NanoDrop 2000, and qualified samples were subjected to WGS on the Illumina NovaSeq 6000 platform.

### 2.6 Bioinformatics analysis of WGS

For genomic analysis, the sequenced reads were assembled using SPAdes (version 3.15.4), and phylogenetic relationships were inferred based on core-genome single nucleotide polymorphism (SNP) analysis. Multilocus sequence typing (MLST) was performed in batch mode using the MLST software (https://github.com/tseemann/mlst), referencing the standard *Pseudomonas aeruginosa* scheme available in the PubMLST database (https://pubmlst.org/paeruginosa/). To illustrate clonal relationships, a minimum spanning tree was constructed from the MLST allelic profiles using PHYLOVIZ software (version 2.1). Resistance and virulence genes were identified by ABRicate (v1.0.1). For resistance genes, thresholds of ≥95% sequence identity and ≥95% coverage were applied. For virulence genes, thresholds of ≥95% sequence identity and ≥90% coverage were applied.

### 2.7 PFGE homology analysis

CRPA isolates and Salmonella serotype Braenderup strain H9812 (used as a molecular size marker) were cultured on LB agar at 37 °C for 18–24 h. Bacterial suspensions were prepared in 600 µL of Cell Suspension Buffer (CSB) and adjusted to an OD_600_ of 1.3-1.4. A 200 µL aliquot of the suspension was mixed with 20 µL of proteinase K (20 mg/mL) and 200 µL of molten 1% Pulsed Field Certified Agarose (PFCA) containing 1% Sodium Dodecyl Sulfate (SDS). The mixture was immediately dispensed into plug molds and allowed to solidify at room temperature for 30 min. The plugs were then transferred to 50 mL tubes containing 5 mL of Cell Lysis Buffer (CLB) supplemented with 25 µL of proteinase K and incubated in a shaking water bath at 54 °C with 170 rpm for 2–3 h. Following lysis, the plugs were washed sequentially with sterile ddH_2_O and TE buffer at 50 °C. The washed plugs were stored in TE buffer at 4 °C until use. Subsequently, the plugs were subjected to restriction enzyme digestion, rinsed with 1 mL of 0.5× TBE, and placed onto the comb teeth. Electrophoresis was performed in 0.5× TBE buffer at 14 °C using a 1% PFCA gel. After electrophoresis, the gel was stained with Gel-Red, destained, and imaged. Cluster analysis was performed using GelJ software, with isolates exhibiting ≥85% similarity defined as the same clone type.

## 3 Results

### 3.1 Source and distribution of CRPA isolates

**Figure 1:**
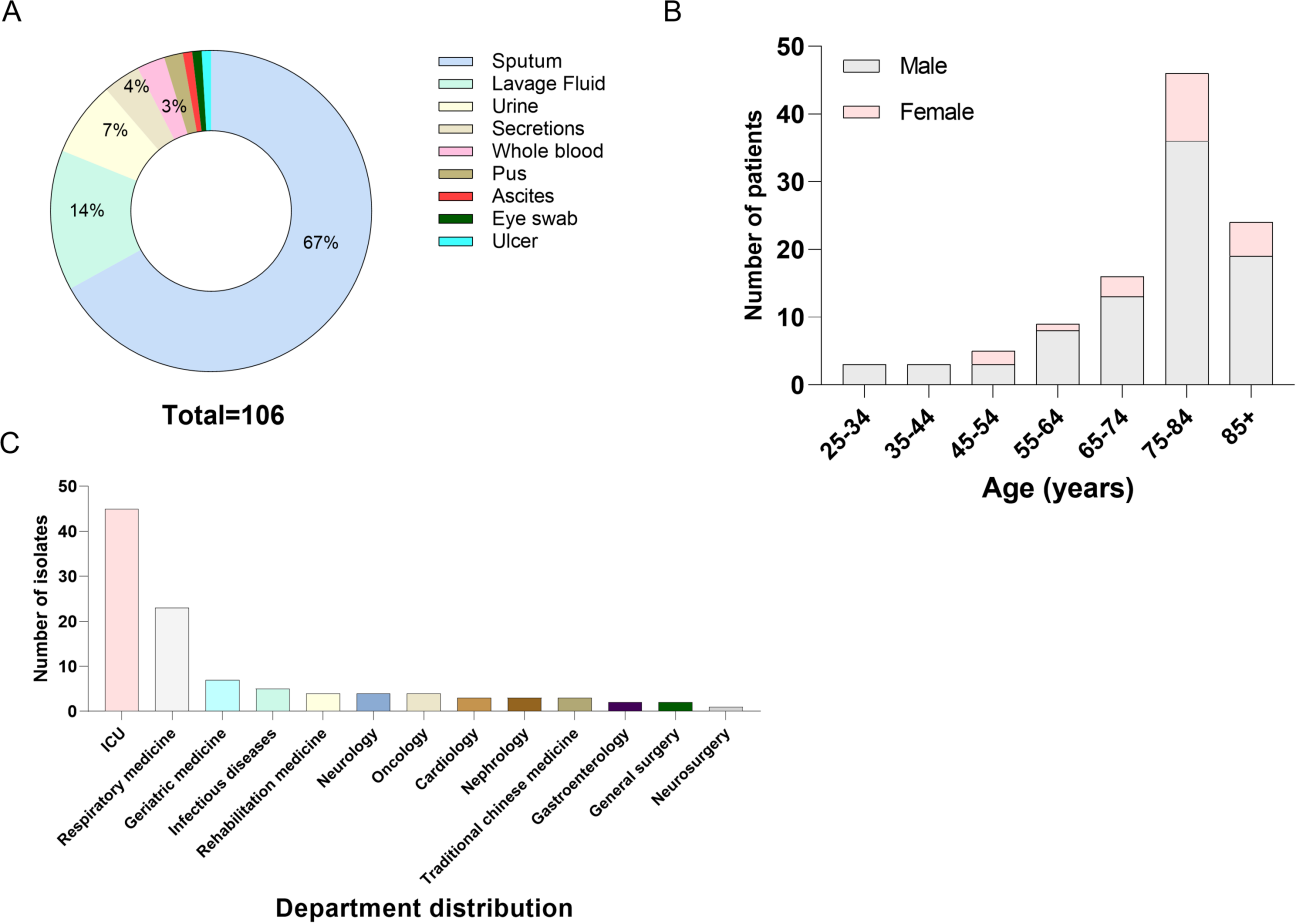
Source and distribution of CRPA isolates. **(A)** Sample types of the 106 CRPA isolates. **(B)** Age and sex distribution of patients infected with the CRPA strains. **(C)** Department distribution of the 106 CRPA isolates.

A total of 106 CRPA isolates were collected. The majority of strains were sourced from the ICU and Respiratory Medicine Department, and the patient population was predominantly elderly males (**Figures 1A, B**). The majority of isolates were obtained from respiratory specimens, including sputum (71 isolates) and bronchoalveolar lavage fluid (15 isolates) (**Figure 1C**).

### 3.2 Antibiotic susceptibility tests

These 106 CRPA isolates were subjected to antimicrobial susceptibility testing using the Vitek 2 Compact automated system. All isolates (100%, 106/106) were completely resistant to imipenem (IPM) and also exhibited 100% resistance to cefazolin (CZ) and cefotetan (CTT) (103/103). A markedly high resistance rate of 97.09% (100/103) was observed for nitrofurantoin (FM). Furthermore, the isolates demonstrated high levels of resistance to most antipseudomonal β-lactams: aztreonam (ATM) at 62.26% (66/106), cefoperazone/sulbactam (SCF) at 56.60% (60/106), ceftazidime (CAZ) at 55.66% (59/106), with piperacillin/tazobactam (TZP) and cefepime (FEP) showing resistance rates approaching 50% (49.06%, 52/106 and 50.00%, 53/106, respectively). Regarding fluoroquinolones, resistance rates to ciprofloxacin (CIP) and levofloxacin (LVX) were 60.38% (64/106) and 54.72% (58/106), respectively. In contrast, aminoglycosides retained favorable antibacterial activity, with susceptibility rates as high as 91.51% (97/106) for amikacin (AN) and 94.17% (97/103) for tobramycin (TM). Detailed susceptibility results are shown in **Table 1**.

**Table 1:** Antimicrobial susceptibility results of the 106 CRPA strains.

Antibacterial drugs	Total	R	Resistance rate (%)	I	Intermediary rate (%)	S	Sensitivity rate (%)
AN	106	6	5.66	3	2.83	97	91.51
TM	103	6	5.83	0	0.00	97	94.17
TZP	106	52	49.06	27	25.47	27	25.47
FEP	106	53	50.00	14	13.21	39	36.79
CAZ	106	59	55.66	15	14.15	32	30.19
SCF	106	60	56.60	16	15.09	30	28.30
CIP	106	64	60.38	7	6.60	35	33.02
LVX	106	58	54.72	12	11.32	36	33.96
ATM	106	66	62.26	14	13.21	26	24.53
FM	103	100	97.09	3	2.91	0	0.00
CZ	103	103	100.00	0	0.00	0	0.00
CTT	103	103	100.00	0	0.00	0	0.00
IPM	106	106	100.00	0	0.00	0	0.00

AN, amikacin; TM, tobramycin; TZP, piperacillin/tazobactam; FEP, cefepime; CAZ, ceftazidime; SCF, cefoperazone/sulbactam; CIP, ciprofloxacin; LVX, levofloxacin; ATM, aztreonam; FM, nitrofurantoin; CZ, cefazolin; CTT, cefotetan, IPM, imipenem.

### 3.3 mCIM

**Figure 2:**
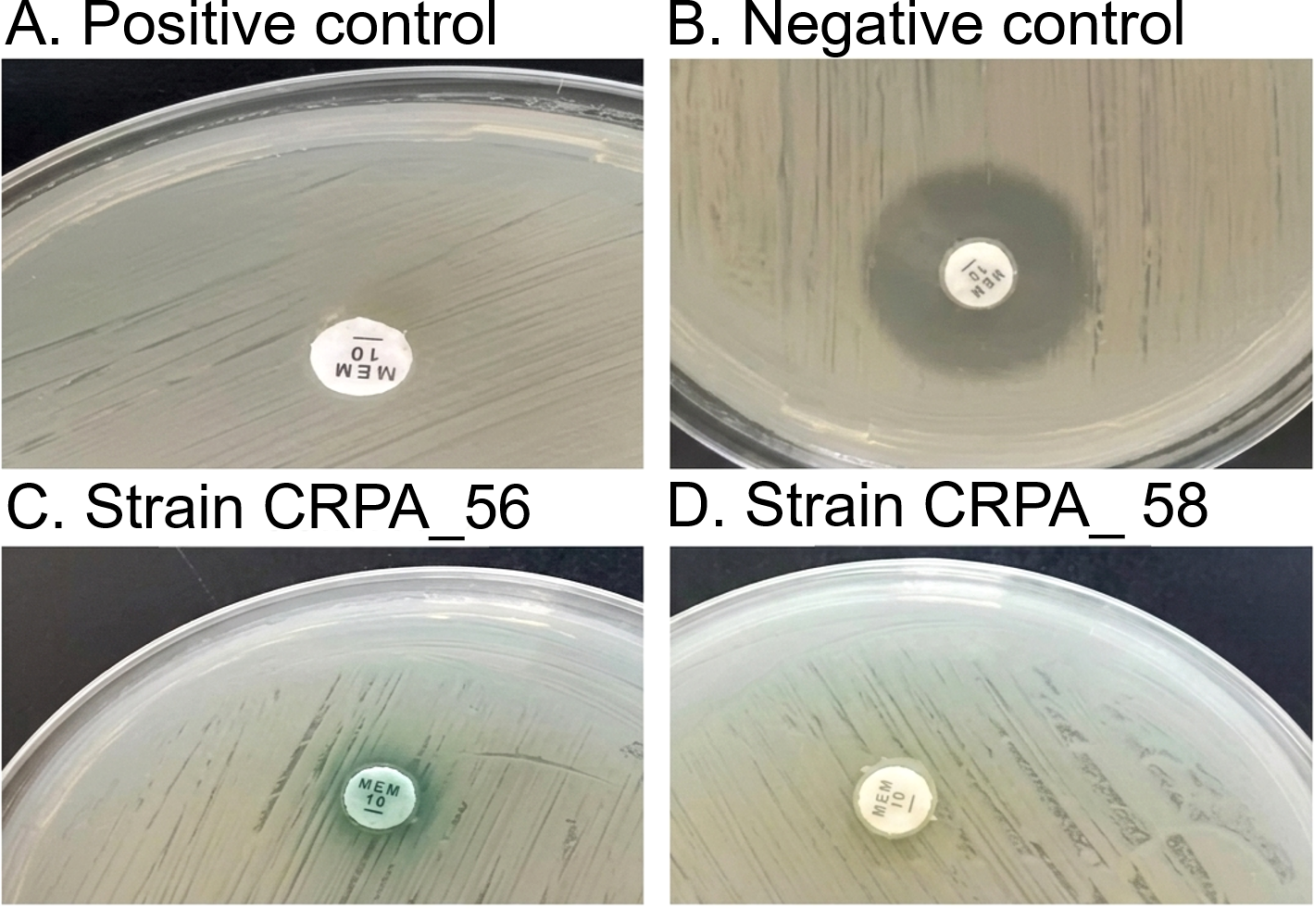
mCIM results of two positive strains. The mCIM assay was performed according to the CLSI guidelines (2018) on 106 clinical isolates of CRPA to evaluate carbapenemase production. Representative phenotypic results are shown. The labels **(A-D)** in the figure indicate: **(A)**, positive control (*Klebsiella pneumoniae* ATCC BAA-1705); **(B)**, negative control (*Klebsiella pneumoniae* ATCC BAA-1706); **(C)**, clinical isolate CRPA_56; **(D)**, clinical isolate CRPA_58. Among the 106 CRPA isolates tested, only CRPA_56 and CRPA_58 were confirmed as carbapenemase-positive.

mCIM was performed according to the CLSI guidelines (2018). Serving as an important supplement to routine antimicrobial susceptibility testing, the mCIM method not only effectively improves the detection rate of carbapenemases in PA but also offers significant advantages including high sensitivity, simple operation, low cost, and no requirement for special instruments or equipment. In this study, the mCIM assay was used to detect carbapenemase production in 106 clinical isolates of CRPA. The results showed that the vast majority of isolates tested negative, with only two isolates (PA56 and PA58) confirmed as carbapenemase-positive (**Figure 2**).

### 3.4 Analysis of *oprD* gene integrity in CRPA strains

OprD is the primary entry channel for carbapenems, particularly imipenem. To determine whether porin alterations contribute to carbapenem resistance in CRPA strains, we performed *oprD* gene amplification assays on all 106 clinical isolates. The results showed that the *oprD* gene remained intact in the vast majority of isolates (100/106, 88.7%), with only 6 isolates (5.7%) exhibiting gene deletion (**Supplementary Figure 1**). This low deletion rate suggests that OprD loss is likewise not a major driver of carbapenem resistance in our CRPA collection.

### 3.5 Genetic characterization of CRPA

**Figure 3:**
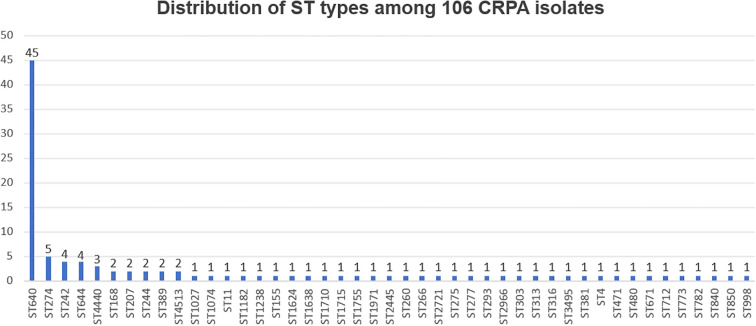
Distribution of ST types among 106 CRPA isolates. The sequence types of all 106 CRPA isolates were determined based on whole-genome sequencing data. The bar graph shows the number of isolates belonging to each ST, arranged from high to low. ST640 was the most prevalent sequence type, accounting for 45 isolates.

To gain deeper insights into the resistance mechanisms of the CRPA isolates, all 106 strains were subjected to WGS and subsequent bioinformatics analysis. Sequence typing based on WGS data showed that ST640 was the most common sequence type, comprising 45 of the 106 isolates (**Figure 3**).

**Figure 4:**
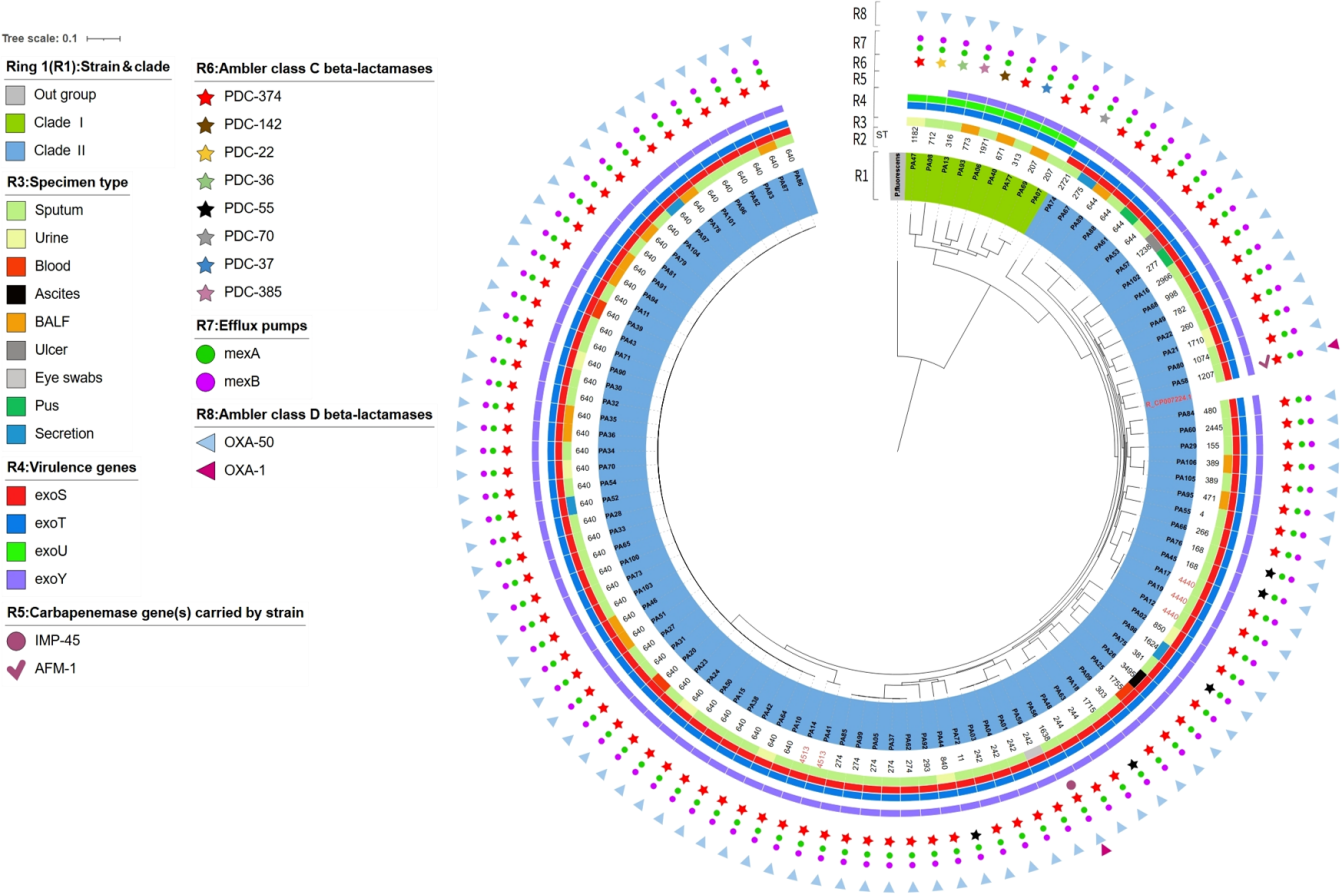
Phylogenetic tree and distribution of antimicrobial resistance and virulence genes among 106 CRPA strains. A maximum-likelihood phylogenetic tree was constructed based on core-genome SNPs. From inner to outer, the concentric layers represent: specimen types, virulence genes, Ambler class B β-lactamases (carbapenem resistance genes), class C β-lactamases, efflux pump genes, and class D β-lactamases.

The sequencing results were highly consistent with the preliminary mCIM phenotypic findings: only two strains, PA56 and PA58, carried carbapenemase genes. The PA56 harbored the B1 subclass metallo-β-lactamase gene blaIMP-45, while PA58 carried the B1 subclass metallo-β-lactamase gene blaAFM-1(**Figure 4**). Regarding non-enzymatic resistance-related genes, all 106 strains carried the efflux pump-associated genes (*mexA*, *mexB*), the AmpC cephalosporinase gene blaPDC, and the class D β-lactamase gene blaOXA-50. Sequence alignment revealed that all blaPDC genes belonged to various subtypes, suggesting considerable genetic diversity of the AmpC enzyme within this strain collection (**Figure 4**).

Analysis of virulence factors showed that all 106 strains carried the *exoT* gene. Regarding the type III secretion system effector protein genes, the carriage rate of *exoY* was 98.1% (104/106), *exoS* was 91.5% (97/106), while *exoU* was only 8.5% (9/106). The *exoS* and *exoU* are generally considered to be distributed in a mutually exclusive manner in *PA*, as reported previously (Feltman et al., 2001; Ozer et al., 2019). Consistent with this paradigm, strains with the *exoS*+/*exoU-* genotype were absolutely predominant in our study (**Figure 4**).

### 3.6 PFGE molecular typing and clonal dissemination analysis

**Figure 5:**
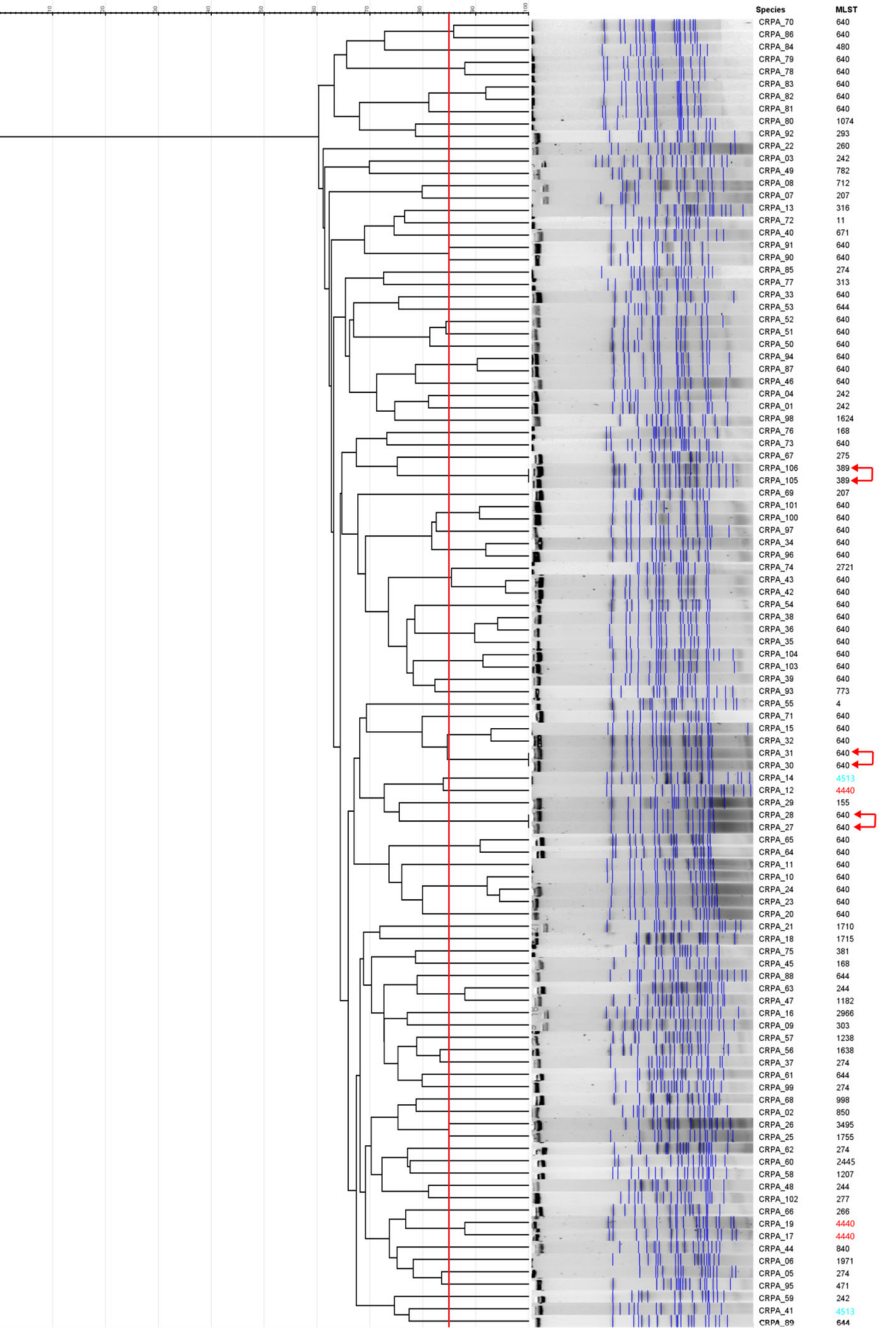
PFGE profiles of the 106 CRPA strains. The PFGE profiles were generated by digesting genomic DNA of the 106 CRPA strains with *Spe*I. Strain IDs and their corresponding ST types are indicated on the left, and the novel ST types ST4440 and ST4513 are highlighted in red and blue, respectively. Arrows denote clusters of strains exhibiting identical PFGE patterns (100% similarity).

PFGE typing demonstrated considerable genetic heterogeneity among the 106 CRPA isolates, with similarity coefficients ranging from 60% to 100%, and a total of 84 distinct patterns were identified (**Figure 5**). Six clusters exhibited 100% pattern identity, but each cluster contained only two isolates, and no cluster comprising more than two isolates was observed. The 45 isolates sharing the same sequence type (ST640) also displayed markedly divergent PFGE profiles, suggesting that ST640 may have undergone local microevolution or genetic differentiation.

### 3.7 Identification of two novel sequence types

Among the 106 CRPA isolates, we found two novel sequence types (ST4440, ST4513) and the information can be checked in the two internet website pages (https://pubmlst.org/bigsdb?page=profileInfo&db=pubmlst_paeruginosa_seqdef&scheme_id=1&profile_id=4440; https://pubmlst.org/bigsdb?page=profileInfo&db=pubmlst_paeruginosa_seqdef&scheme_id=1&profile_id=4513). ST4440 comprised three isolates (CRPA_12,CRPA_17,CRPA_19). ST4513 comprised two isolates (CRPA_14,CRPA_41). All five isolates were negative for *exoU* and positive for *exoS*, *exoT*, and *exoY*. Resistance gene analysis revealed that all isolates harbored PDC-374. Detailed information is shown in **Table 2**. PFGE analysis revealed that the three ST4440 isolates exhibited markedly divergent patterns among themselves, as did the two ST4513 isolates, indicating that these isolates had all undergone genetic differentiation rather than recent clonal transmission (**Figure 5**).

**Table 2:** Characteristics of the two novel sequence types.

Strain No.	CRPA_12	CRPA_17	CRPA_19	CRPA_14	CRPA_41
ST type	4440	4440	4440	4513	4513
Specimen type	Sputum	Sputum	Sputum	Sputum	Sputum
Sex	Male	Male	Male	Female	Female
Age	90	91	66	92	94
exoS	+	+	+	+	+
exoT	+	+	+	+	+
exoU	–	–	–	–	–
exoY	+	+	+	+	+
blaPDC	PDC-374	PDC-374	PDC-374	PDC-374	PDC-374
AN	S	S	S	S	S
TM	S	S	S	S	S
TZP	R	R	R	I	R
FEP	I	I	S	S	I
CAZ	R	I	S	S	R
SCF	R	R	R	I	R
CIP	R	R	S	I	R
LVX	R	R	S	I	R
ATM	I	R	I	R	R
FM	R	R	R	R	R
CZ	R	R	R	R	R
CTT	R	R	R	R	R
IPM	R	R	R	R	R
Co-isolated bacteria	–	–	–	MRSA	MRSA
Hospitalization days	18	17	32	20	12
Antibiotic therapy	LVX MEM	CZ MEM	TZP LVX MEM	LVX MEM VAN	Piperacillin/ sulbactam VAN
Outcome	Improved	Improved	Improved	Improved	Died

MRSA, Methicillin-resistant *Staphylococcus aureus*; MEM, meropenem; VAN, vancomycin.

### 3.8 Clinical characteristics and patient outcomes

#### 3.8.1 Overall clinical characteristics of the 106 patients with CRPA isolates

To provide a clearer clinical context for the CRPA isolates, the baseline characteristics of the 106 patients were further summarized in **Table 3**. Pulmonary infection was the predominant clinical presentation (87.7%), and 42.5% of patients were admitted to the ICU. Prior carbapenem exposure was observed in 40.6% of cases. The overall mortality rate was 18.9%, whereas 80.2% of patients showed clinical improvement or cure. These findings indicate that CRPA infections in this cohort were mainly associated with elderly, hospitalized patients with severe underlying conditions.

**Table 3:** Clinical characteristics of the 106 patients with CRPA infection.

Characteristic	Value
Total patients	106
Male sex	85 (80.2%)
Female sex	21 (19.8%)
Age, years	79 (IQR, 65–85)
ICU admission	45 (42.5%)
Pulmonary infection	93 (87.7%)
Prior carbapenem exposure	43 (40.6%)
Improved or cured	85 (80.2%)
Death	20 (18.9%)
Length of stay, days	29

#### 3.8.2 Patients with carbapenemase-producing isolates CRPA_56 and CRPA_58

For the two carbapenemase-producing isolates, CRPA_56 carrying blaIMP-45 was recovered from a 50-year-old female patient with pulmonary infection, while CRPA_58 carrying blaAFM-1 was isolated from an 88-year-old male patient with pulmonary infection. Upon admission, CRPA_56 received empirical levofloxacin, later switched to aztreonam based on susceptibility testing results, and the patient’s symptoms relieved. CRPA_58 was initially given empirical ceftriaxone; however, after six days without significant improvement in cough and sputum production, levofloxacin was added per susceptibility testing, and the patient’s symptoms improved markedly. Both patients had no prior carbapenem exposure, relatively short hospital stays (10 and 17 days), and favorable clinical outcomes. These findings suggest that carbapenemase-producing CRPA isolates were rare in this cohort and were not associated with worse clinical outcomes, supporting the observation that carbapenem resistance in this setting is predominantly driven by non-enzymatic mechanisms rather than carbapenemase production.

#### 3.8.3 Patients with two novel sequence types ST4440 and ST4513

The three patients infected with ST4440 isolates were all male. All three patients had type 2 diabetes mellitus. Among them, two patients had hypertension, urinary tract infection, pulmonary infection, benign prostatic hyperplasia and permanent pacemaker implantation. All three patients received meropenem-containing regimens and achieved clinical improvement, with hospital stays ranging from 17 to 32 days. In contrast, the two patients infected with ST4513 isolates were female. Both patients shared a similar spectrum of chronic diseases: metabolic (type 2 diabetes mellitus), cardiopulmonary (hypertension, cardiovascular disease, chronic bronchitis), neurological (vascular dementia, epilepsy with tonic-clonic seizures, subcortical arteriosclerotic encephalopathy), and skeletal metabolic (osteoporosis) disorders. In addition, both patients had concurrent MRSA infection and received vancomycin-containing therapy. Their hospital stays were 20 and 12 days, respectively. One patient improved, while the other died. Antimicrobial susceptibility testing showed that all five isolates were resistant fosfomycin (FM), cefazolin (CZ), cefotetan (CTT), with minor variations in susceptibility to other agents. Detailed information is shown in **Tables 2**, **4**.

**Table 4:** Underlying diseases of patients with the two novel ST types.

CRPA_12	CRPA_17	CRPA_19	CRPA_14	CRPA_41
Pulmonary infection; UTI; T2DM; Hypertension; Post pacemaker implantation for sick sinus syndrome; BPH;	Chronic obstructive pulmonary disease with pulmonary infection; UTI; T2DM; Hypertension Post pacemaker implantation for sick sinus syndrome; BPH; Mild anemia;	Decompensated liver cirrhosis; Calculous cholecystitis; T2DM;	Coronary atherosclerotic heart disease; Acute exacerbation of chronic bronchitis; Hypertension; T2DM; Vascular dementia; Osteoporosis Epilepsy with tonic-clonic seizures Subcortical arteriosclerotic encephalopathy	Coronary atherosclerotic heart disease; Acute exacerbation of chronic bronchitis Hypertension; T2DM; Vascular dementia Osteoporosis Epilepsy with tonic-clonic seizures Subcortical arteriosclerotic encephalopathy

UTI, Urinary tract infection; T2DM, Type 2 diabetes mellitus; BPH, Benign prostatic hyperplasia.

## 4 Discussion

PA is an important opportunistic pathogen capable of causing both endogenous and exogenous infections. The World Health Organization has classified CRPA as a high‑priority pathogen in its 2024 Bacterial Priority Pathogens List. (Sati et al., 2025). Therefore, an in‑depth understanding of the molecular epidemiological characteristics of CRPA is of great significance for guiding the development of new antibiotics and improving clinical infection prevention and control.

Antimicrobial susceptibility testing of the 106 CRPA isolates revealed complete resistance (100%) to imipenem in all strains. Among the 103 isolates tested against cefazolin and cefotetan, complete resistance was also observed. High resistance rates were noted for most other β-lactams (49%–62%). Fluoroquinolone resistance exceeded 54%, while aminoglycosides retained excellent activity, with susceptibility rates greater than 91% for both amikacin and tobramycin. PFGE classified the isolates into 84 pulsotypes, demonstrating high genetic diversity with no predominant clonal cluster. Phylogenetic analysis of WGS data showed that 45 strains belonged to ST640, with two novel sequence types, ST4440 and ST4513, identified for the first time. Regarding virulence genes, all strains carried *exoT*, with carriage rates of 98.1% for *exoY* and 91.5% for *exoS*, while *exoU* was present in only 8.5% of strains. In the exploration of the resistance mechanisms of the strains, the sequencing results were highly consistent with the phenotypic mCIM assay, with carbapenemase genes detected in only two strains, PA56 (bla*IMP*-45) and PA58 (bla*AFM*-1). Analysis of non-enzymatic resistance mechanisms revealed a low deletion rate (5.7%, 6/106) of the gene encoding the OprD porin, whereas all strains (100%, 106/106) carried efflux pump-related genes (*mexA*, *mexB*) and the AmpC cephalosporinase gene (bla*PDC*).

WGS provided critical information beyond conventional AST, mCIM, and PFGE. AST only yielded phenotypic resistance profiles, and mCIM detected carbapenemase production phenotypically but could not identify specific genes. In contrast, WGS pinpointed the exact carbapenemase genes and revealed that all isolates carried a repertoire of non-enzymatic resistance genes, indicating that non-enzymatic resistance predominates in this cohort. Regarding virulence, WGS characterized the T3SS effector genes *exoT*, *exoY*, *exoS*, and *exoU*. For genetic relatedness, PFGE showed high heterogeneity (84 distinct pulsotypes), whereas WGS identified ST640 as a potentially dominant clone and uncovered microevolution within this clone that PFGE could not resolve.

The above WGS-based findings indicate that, among carbapenemase-negative CRPA isolates, the synergy between efflux pump overexpression and AmpC enzyme hyperproduction may serve as the dominant mechanism driving carbapenem resistance in this region. This finding is consistent with the conclusions of Dantas RCC et al (Dantas et al., 2017). Their study demonstrated that in non-metallo-β-lactamase-producing isolates, the combination of multiple intrinsic resistance mechanisms may collectively contribute to the MDR/XDR phenotype, primarily characterized by AmpC hyperproduction, OprD porin loss, and elevated expression of the MexAB-OprM and MexXY efflux pumps (Dantas et al., 2017). Consistently, a meta-analysis of PA strains from Iran identified the coexistence of these same mechanisms, particularly *oprD* downregulation, efflux pump oprM expression, and AmpC hyperproduction, is a critical factor driving the emergence of MDR and XDR strains (Alimoghadam et al., 2024). Further mechanistic insight into this synergy is provided by a study showing that MexAB-OprM and AmpC have a cumulative effect on β-lactam resistance when co-upregulated (Grosjean et al., 2021). This research clarifies that while MexAB-OprM alone is insufficient to raise MICs above clinical breakpoints, its synergistic interaction with AmpC plays a decisive role in the evolution of high-level resistance to key antipseudomonal agents, including ceftazidime, cefepime, and meropenem (Grosjean et al., 2021). And a significant polymorphic distribution of the bla*PDC* gene was observed. Given that the expression of the AmpC enzyme and efflux pump-related genes (mexA, mexB) may constitute one of the key mechanisms underlying PA resistance to β-lactam antibiotics in this region, further in-depth molecular investigations are warranted to elucidate whether functional differences exist among distinct bla*PDC* subtypes and whether these variations directly impact the resistance levels of strains to carbapenems.

Regarding the class D β-lactamase gene blaOXA-50, although existing studies indicate that it alone exhibits weak hydrolytic activity against carbapenems (e.g., imipenem) (Girlich et al., 2004), its potential for evolution and synergistic interaction with other resistance mechanisms should not be overlooked. A 2022 kinetic analysis revealed that blaOXA-50 variant, OXA-488, hydrolyzes imipenem with a catalytic efficiency twice that of the wild-type enzyme (Streling et al., 2022). Another study indicated that the coexistence of blaOXA-50 with crpP resistance gene is closely associated with the difficult-to-treat resistance (DTR) phenotype (Jin et al., 2025). The widespread coexistence of blaOXA-50 with blaPDC, mexA, and mexB observed in this study highlights the need for further investigation to determine whether this genetic background contributes to carbapenem resistance. Furthermore, in strains harboring these genes along with OprD porin deficiency, it remains to be determined whether resistance levels are significantly altered and whether this convergence of multiple mechanisms further complicates clinical treatment. Addressing these questions will contribute to a more comprehensive understanding of the evolutionary mechanisms underlying carbapenem resistance in CRPA.

This study has several limitations. First, it was conducted at a single center, which may limit the generalizability of the findings to other regions or hospital settings. Second, although resistance- and virulence-associated genes were identified by WGS, no gene expression analyses were performed to confirm their transcriptional activity. Third, the assessment of *oprD* was limited to PCR-based detection of gene integrity, and no further functional validation was conducted. Finally, we did not perform a direct analysis of the relationship between genotype and treatment outcomes. Further multicenter studies integrating genomic, functional, and clinical outcome data are needed to better clarify the resistance mechanisms and clinical significance of CRPA.

## Data Availability

The original contributions presented in the study are included in the article/supplementary material. Further inquiries can be directed to the corresponding author.

## Supplementary material

Supplementary Figure 1 PCR identification of the oprD gene. Strains CRPA_3, CRPA_4, CRPA_8, CRPA_58, CRPA_75, and CRPA_77 are *oprD* absent strains (no PCR products). Positive control (+): *K. pneumoniae* BAA-1705; negative control (-): *K. pneumoniae* BAA-1706.
