# Supplementary figures and images for: Whole-genome sequencing reveals molecular characterization of carbapenem-resistant Pseudomonas aeruginosa clinical isolates from a third-tier general hospital in southwest China
Source: Front Cell Infect Microbiol. 2026 May 8;16:1843484. doi: 10.3389/fcimb.2026.1843484 (PMC13194390; doi:10.3389/fcimb.2026.1843484)

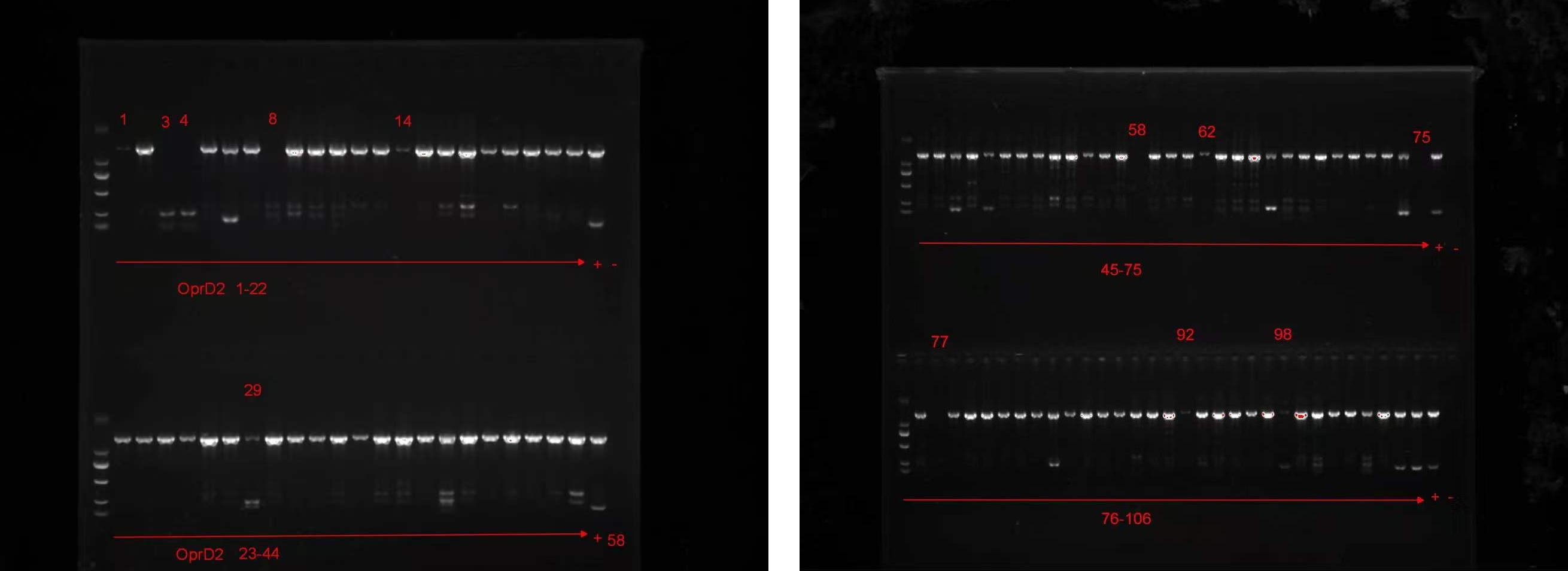

Supplement: Supplementary Figure 1 — PCR identification of the oprD gene. Strains CRPA_3, CRPA_4, CRPA_8, CRPA_58, CRPA_75, and CRPA_77 are oprD absent strains (no PCR products). Positive control (+): K. pneumoniae BAA-1705; negative control (-): K. pneumoniae BAA-1706. [file Image1.jpeg]
